# Tumor-associated macrophages modulate resistance to oxaliplatin via inducing autophagy in hepatocellular carcinoma

**DOI:** 10.1186/s12935-019-0771-8

**Published:** 2019-03-25

**Authors:** Xiu-Tao Fu, Kang Song, Jian Zhou, Ying-Hong Shi, Wei-Ren Liu, Guo-Ming Shi, Qiang Gao, Xiao-Ying Wang, Zhen-Bin Ding, Jia Fan

**Affiliations:** 1 Department of Liver Surgery & Transplantation, Liver Cancer Institute, Zhongshan Hospital, Fudan University, 1609 Xietu Road, Shanghai, 200032 China; 2 Key Laboratory for Carcinogenesis and Cancer Invasion, Chinese Ministry of Education, Shanghai, China

**Keywords:** Hepatocellular carcinoma, Tumor associated macrophage, Autophagy, Chemo-resistance, Oxaliplatin

## Abstract

**Background:**

Oxaliplatin-based chemotherapy is widely used to treat hepatocellular carcinoma (HCC). Recent studies suggested that therapeutic resistance of tumors was affected by tumor microenvironment (TME). As a major component of TME, the role of tumor-associated macrophages (TAMs) on drug resistance in HCC is largely unknown.

**Methods:**

26 HCC samples were obtained from patients who had underwent transarterial chemoembolization (TACE) within 3 months before receiving curative resections. Immunohistochemistry was applied to detect the density of TAMs in these tissues. SMMC-7721 and Huh-7 cell lines were used to co-culture with THP-1 derived macrophages. Under oxaliplatin treatment, cell death was measured using MTT and annexin V/propidium iodide assays. Autophagy activation was evaluated by GFP-LC3 redistribution and LC3 conversion in SMMC-7721 and Huh-7. Short-interfering RNA against ATG5 gene was applied to inhibit autophagy. In vivo validation was conducted in Huh-7 with or without macrophages using an HCC xenograft model in nude mice after oxaliplatin administration.

**Results:**

We found that the density of TAMs in HCC samples was associated with the efficacy of TACE. Macrophages inhibited cell death induced by oxaliplatin in HCC cells. Autophagy was functionally activated in HCC cells after co-culturing with macrophages. Suppression of autophagy using RNA interference of ATG5 in HCC cells promoted the oxaliplatin cytotoxicity in the co-culture system. Critically, co-implantation with macrophages in HCC xenografts weakens cytotoxic effect of oxaliplatin through inducing autophagy to avoid apoptosis.

**Conclusions:**

Our results suggest that TAMs induce autophagy in HCC cells which might contribute to oxaliplatin resistance. Targeting TAMs is a promising therapeutic strategy to enhance the effects of chemotherapy oxaliplatin in HCC patients.

## Background

Hepatocellular carcinoma (HCC) is a major malignancy and the second leading cause of cancer death worldwide [1, 2]. Due to locally advanced or metastatic disease, the prognosis of HCC is quite poor, and surgery in combination with adjuvant chemotherapy provides a long-term survival possibility for HCC patients [3]. With the development of regional cancer therapy and multimodality treatments, localized unresectable large HCCs have been transformed to resectable small HCCs [4]. Transarterial chemoembolization (TACE) is used widely for unresectable HCCs and has been recommended as a standard loco-regional palliative treatment for these patients [5]. However, not all patients received the same beneficial effect [6].

Oxaliplatin is one of the first-line drugs used in TACE, and it is widely used in the chemotherapeutic regimes to reduce HCC recurrence and prolong survival. However, due to the development of cellular resistance, its efficacy is limited, which is still a difficult problem for the successful chemotherapy of HCC [7, 8].

Recent studies suggested that the therapeutic resistance of tumors relies on extrinsic mechanisms represented by the cross-talk between tumor cells and other cellular components of the tumor microenvironment (TME), in particular immune cells [9]. Tumor-associated macrophages (TAMs) constitute the dominant myeloid cell population in many tumors and play a key role in multiple aspects of TME, including therapeutic resistance [10–12]. In our previous study, we demonstrated that TAMs-secreted IL-8 could induce epithelial-mesenchymal transition in HCC cells by activating the JAK2/STAT3/Snail pathway [13]. It is also reported that the TAMs density positively correlated with poor prognosis in several types of solid tumor [12, 14], including HCC [15, 16]. There is a growing body of evidence that TAM and its products are involved in modulating behavior of cancer cells in chemotherapy [17–19]. However, the relationship between TAMs and chemo-resistance in HCC is still obscure.

In our previous study, it was revealed that autophagy activation in HCC could contribute to the tolerance of oxaliplatin via reactive oxygen species (ROS) modulation [20]. Interactions between the microenvironment and autophagy have also previously been reported [21–23]. Thus, we presumed that TAMs may induce resistance to oxaliplatin via activating autophagy in HCC cells.

## Materials and methods

### Patient samples

Patient samples were collected after obtaining informed consent, according to an established protocol approved by the Ethics Committee of Zhongshan Hospital, Fudan University. The data collected does not contain any information that could identify the patients. We collected information on HCC patients who had underwent TACE within 3 months prior to radical resections at the Liver Cancer Institute, Zhongshan Hospital, Fudan University, in 2016. The diagnosis of HCC was confirmed by histological examination. Patients were divided into two groups, according to the tumor growth after receiving TACE (tumor shrinkage vs. tumor non-shrinkage). The tumor size was measured by computed tomography or nuclear magnetic resonance imaging. 9 patients were elected to the tumor shrinkage group and 17 patients were elected to the tumor non-shrinkage group for further research.

### Immunohistochemistry

Immunohistochemistry was performed with rabbit anti-human CD68 (1:1000, ab125212, Abcam, Cambridge, UK), using a two-step protocol (Novolink Polymer Detection System, Novocastra, Newcastle, UK), as described previously [24]. Briefly, after microwave antigen retrieval, tissues were incubated with primary antibodies for 60 min at room temperature and then incubated with secondary antibody (RE7112, Novolink Polymer, Newcastle, UK) for 30 min. The sections were developed in 3,3′-diaminobenzidine solution under microscopic observation and counterstained with hematoxylin. Negative control slides, in which the primary antibodies were omitted, were included in all assays. In each section, staining was captured by Leica QWin Plus version 3 software (Leica Microsystems, Wetzlar, Germany).

### Cell preparations and compounds

The human HCC cell lines Huh-7, SMMC-7721 and the human monocyte leukemia cell line THP-1 were purchased from the Institute of Biochemistry and Cell Biology, Chinese Academy of Sciences, Shanghai, China. The 2 HCC cell lines were cultured in Dulbecco’s Modified Eagle Medium (Invitrogen, Carlsbad, CA, USA), and THP-1 cells were cultured in RPMI 1640 Medium (Invitrogen, Carlsbad, CA, USA). Media were supplemented with 10% heat-inactivated fetal bovine serum (FBS) and 100 U/mL penicillin, and 100 mg/mL streptomycin (Invitrogen, Carlsbad, CA, USA). All cell lines were cultured the cells at 37 °C in a humidified atmosphere of 5% CO_2_.

THP-1 cells were seeded into the upper insert of a 6-well Transwell apparatus (0.4 μm pore size, Corning, Lowell, MA, USA) and treated them with Phorbol myristyl acetate (PMA) (320 nM, P1585, Sigma-Aldrich, St. Louis, MO, USA) for 24 h to obtain PMA-treated macrophages.

Oxaliplatin was purchased from Sigma-Aldrich and dissolved in 100% dimethyl sulfoxide and diluted with DMEM to the desired concentration with a final dimethyl sulfoxide concentration of 0.1% for the in vitro studies.

### Establishment of co-culture system with macrophages and HCC cells

After a thorough wash, PMA-treated THP-1 macrophages seeded in upper inserts were co-cultured with HCC cells which were seeded in a 6-well plate (2 × 10^5^ cells per well) without direct contact. After 24 h of co-culture, the upper inserts containing the macrophages were discarded, and HCC cells were washed and used for subsequent experiments.

### Assessment of cell viability in vitro

MTT (3-(4,5-dimethyl-2-thiazolyl)-2,5-diphenyl-2-H-tetrazolium bromide) kit (Trevigen, Gaithersburg, MD, USA) was used to assess the cell proliferation, according to the manufacturer’s protocol. Cells (5 × 10^3^) were plated in 96-well plates, incubated for 24 h at 37 °C, and treated with the specified agents at defined time points. Annexin V- Alexa Fluor 488 assay (Invitrogen, Carlsbad, CA, USA) and propidium iodide (PI) were applied to determine the number of apoptotic cells, according to the manufacturer’s protocol [25]. Cells were analyzed using a flow cytometer, and data were analyzed using CellQuest software version 3.3 (BD Bioscience, Franklin Lakes, NJ, USA).

### Autophagy analysis

The movement of autophagosomes was detected by GFP label. Huh-7 and SMMC-7721 were transfected with a lentivirus plasmid vector containing GFP-LC3 (C3006, Beyotime, Shanghai, China) which targets autophagosomes. Autophagy activity was assessed using GFP-LC3 redistribution and LC3 conversion. Redistribution of GFP-LC3 was detected and the images were captured by using an inverted fluorescence microscope (Carl Zeiss, Dresden, Germany). The average numbers of GFP-LC3—positive dots per cell were determined in 3 independent experiments. Eight randomly selected fields representing 200 cells were counted. For the LC3 conversion assay, cells were lysed with M-PER Mammalian Protein Extraction Reagent (78501, Pierce, MA, USA) and then subjected to westernblot analysis with an antibody against LC3.

### RNA interference

Autophagy-related 5 homolog (ATG5) RNA interference was accomplished by transfection of HCC cells with specific small interfering RNA (siRNA) duplexes. Primers for the ATG5 targeting sense and the negative control sense were purchased from GenePharma Company (Shanghai, China). SiRNAs were transfected using the Lipofectamine 2000 transfection reagent (Invitrogen, Carlsbad, CA, USA). Cells were lysed 72 h after transfection, and protein was assayed by western blot analysis.

### Western blot analysis

To determine the levels of protein expression, cells were harvested and gently washed with cold phosphate-buffered saline (PBS). Total protein was extracted using RIPA Cell Lysis Buffer (Beyotime, Shanghai, China). For each sample, 50 μg of protein was separated by standard SDS-PAGE and then transferred to polyvinylidene difluoride membranes. The membranes were washed and blocked by 5% nonfat milk powder in TBST buffer for 1 h, and then incubated with specific primary antihuman antibodies against ATG5 (1:1000, #2630, CST, Beverly, MA, USA), LC3B (1:1000, #3868, CST, Beverly, MA, USA) and GAPDH (1:10,000, AP0063, BioWorld, St. Louis Park, MN, USA). After that, the membrane was incubated with horseradish peroxidase–conjugated secondary antibodies. The protein bands were visualized using enhanced chemiluminescence western blotting substrate (Pierce, MA, USA), and captured by ChemiDoc™ XRS + system (Bio-Rad Laboratories, CA, USA) and the densitometry of the protein bands were determined by ImageLab version 3.0 (Bio-Rad Laboratories, CA, USA).

### In vivo tumorigenicity

Huh-7 (5 × 10^6^) and Huh-7 with THP-1 derived macrophages (5 × 10^6^, respectively) were suspended in 100 μL serum-free DMEM and Matrigel (BD Biosciences, CA, USA) (1:1), and then inoculated into the subcutaneous region of the right upper abdomen of nude mice, as described previously [13]. 3 days later, all the mice were injected intraperitoneally with oxaliplatin 5 mg/kg twice a week. The mice were sacrificed 5 weeks after tumor implantation. At autopsy, the volumes of the largest (a) and smallest (b) tumors were measured and the tumor volume was calculated as: V = a × b^2^ × π/6.

### Immunohistochemical staining for LC3 expression and hematoxylin and eosin staining in tissues

Xenografts of Huh-7 with THP-1 derived macrophages were fixed with 4% neutral paraformaldehyde. Next, the paraffin-embedded sections (4 μm in thickness) were prepared for hematoxylin and eosin (H&E) staining and immunohistochemical staining of LC3 (ab128025, Abcam, Cambridge, UK). The steps of immunohistochemical staining were described above. The pretreatment of H&E staining was basically the same as the immunohistochemical steps. Sections were treated with hematoxylin reagent for 5 min after deparaffinization and rehydration and then treated with 1% acid–ethanol for 1 s. Subsequently, the sections were stained by eosin reagent for 3 min. The slides were dehydrated and mounted then photographed by microscopy.

### TUNEL staining assay for cell apoptosis in vivo

Terminal deoxynucleotidyl transferase-mediated deoxyuridine triphosphate nick-end labeling (TUNEL) staining was performed using an In Situ Apoptosis Detection Kit (VB-4005, GeneCopoeia, Rockville, MD, USA), according to the manufacturer’s instructions. Briefly, the tumor slides were incubated with terminal deoxynucleotide transferase (TdT) and a biotinylated nucleotide mixture at 37 °C for 30 min. Subsequently, the endogenous peroxidases was blocked by immersing the slides in 0.3% hydrogen peroxide in PBS for 3–5 min at room temperature. The slides were incubated with streptavidin-HRP and visualized with DAB. Negative controls were set up by substituting distilled water for TdT in the working solution. The results are presented as the ratio of the TUNEL-positive cells to the total number of cells.

### Statistical analysis

Statistical analysis was performed using SPSS 22.0 software (IBM, Chicago, IL, USA). Means were compared between two groups using unpaired, two-tailed Student’s *t* test. The cut-off for statistical significance was *P *< 0.05.

## Results

### TAMs correlate with TACE-resistance in HCC patients

**Fig. 1:**
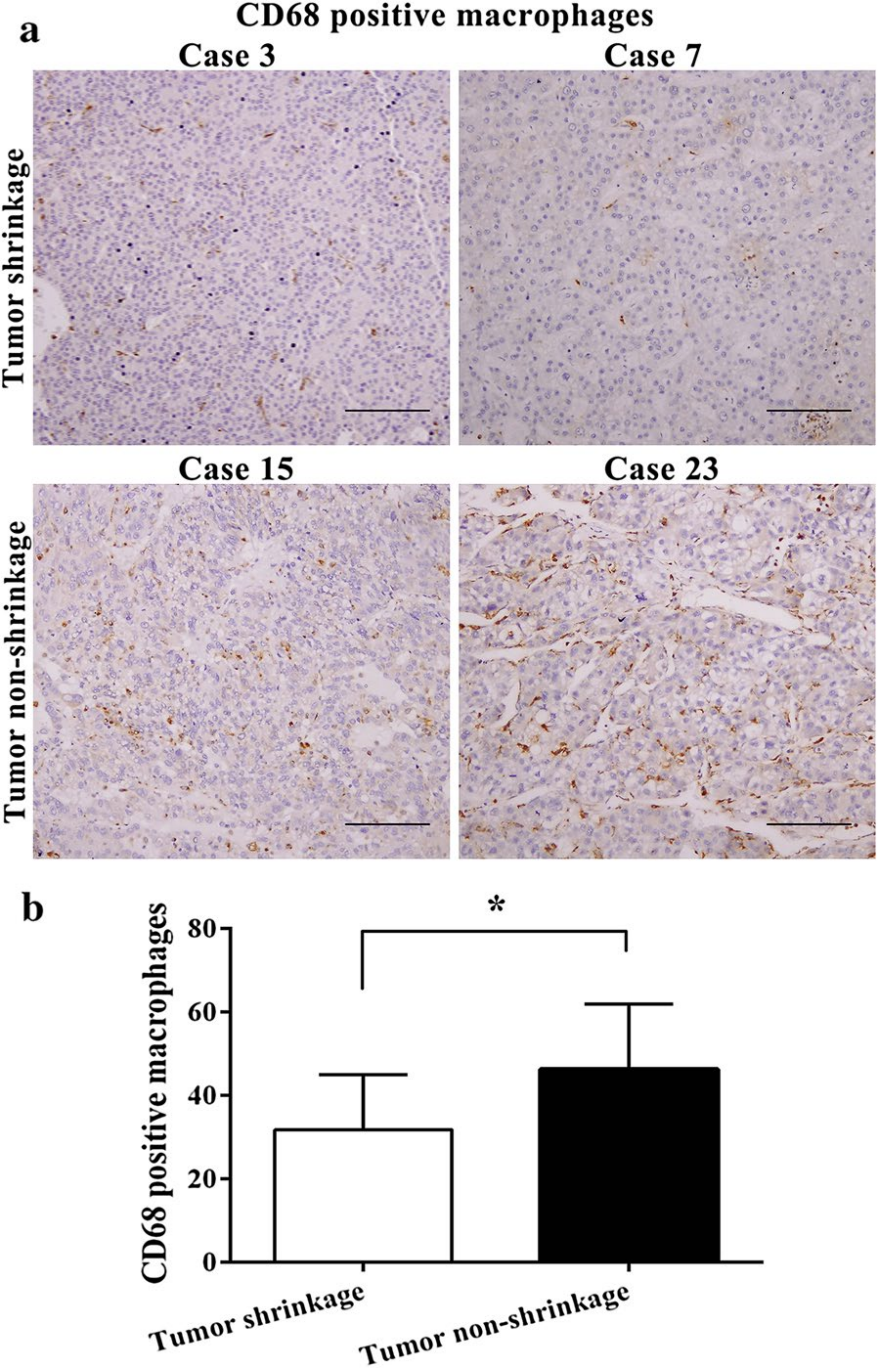
TAMs correlate with TACE-resistance in HCC patients. **a** The macrophages in HCC tissues was assessed by CD68 staining. CD68 positive macrophages were counted in eight random fields (×200). **b** The count of macrophages in tumor shrinkage group was significantly smaller compared to tumor non-shrinkage group. Data shown are mean (SD) from at least 3 independent experiments. Means were compared between two groups using unpaired, two-tailed Student’s t-test. * P < 0.05. Scale bars, 100 μm

The density of macrophages in 26 HCC tissue samples that received preoperative TACE within 3 months were included in our study. CD68 positive macrophages were counted in eight random fields (×200) of every immunohistochemistry section (Fig. 1). Compared with tumor shrinkage group (9 cases), the number of macrophages infiltrated in HCC tissues was significantly increased in tumor non-shrinkage group (17 cases) (31.78 ± 13.24 vs. 46.29 ± 15.66, *P *= 0.027).

### Co-culturing HCC cells with macrophages enhance oxaliplatin-resistance in HCC cells

**Fig. 2:**
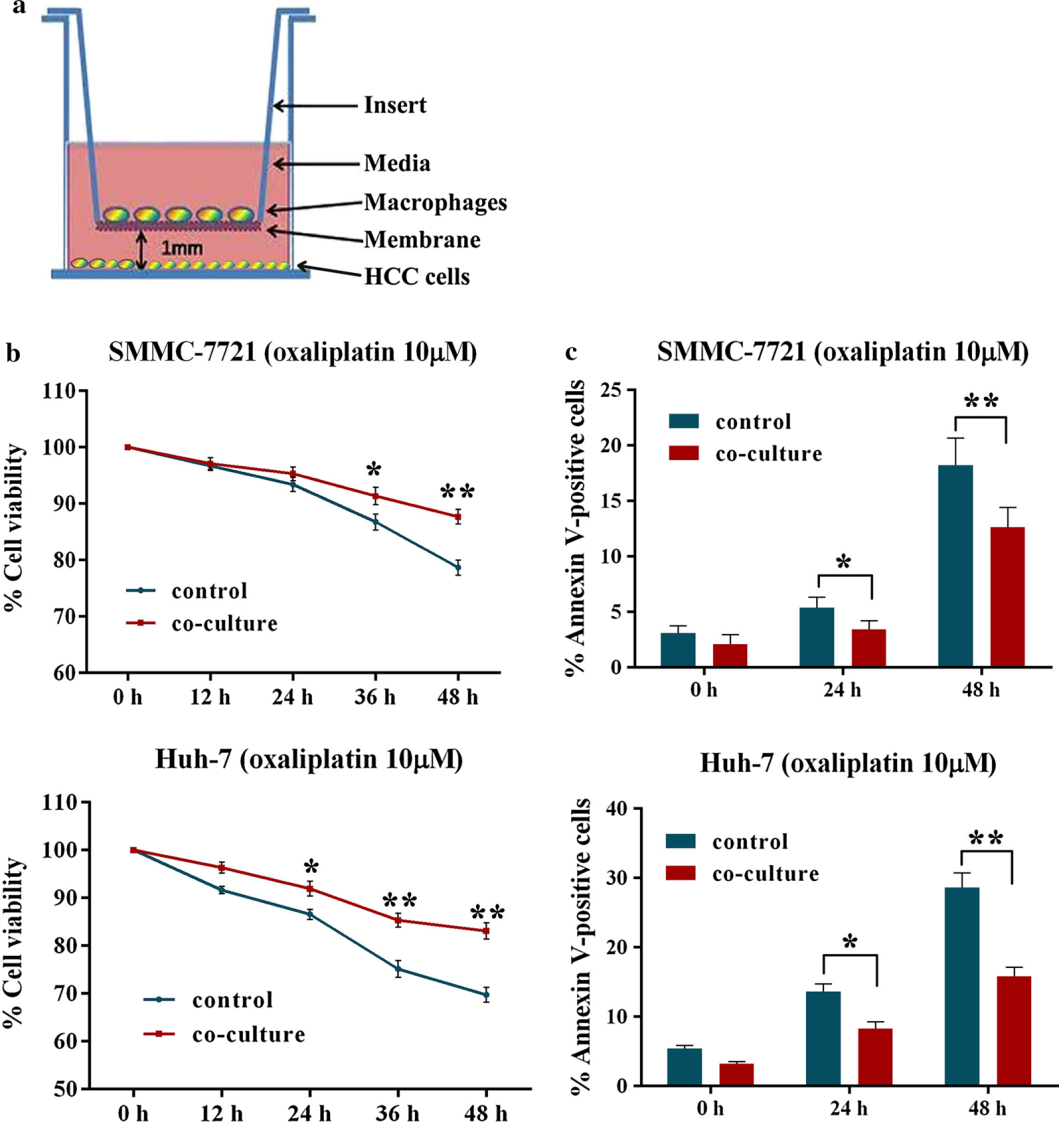
Co-culturing with macrophages enhance oxaliplatin-resistance in HCC cells. **a** HCC cells were co-cultured with macrophages by Transwell system. **b** SMMC-7721 and Huh-7 cell viability was investigated by MTT assay. **c** The percentage of apoptotic SMMC-7721 and Huh-7 cells was determined with Annexin V staining assay. Data shown are mean (SD) from at least 3 independent experiments. Means were compared between two groups using unpaired, two-tailed Student’s t-test. * P < 0.05, ** P < 0.01

SMMC-7721 and Huh-7 cells were co-cultured with PMA-treated THP-1 macrophages in a non-contact Transwell system for 24 h, respectively (Fig. 2a). Then, the macrophages were discarded, and HCC cells were washed and incubated with oxaliplatin. To investigate whether the resistance to oxaliplatin was affected by macrophages, MTT assay was adopted to evaluate the proliferation of SMMC-7721 and Huh-7 cells in response to 10 μM oxaliplatin treatment for 12–48 h. As indicated in Fig. 2b, co-culturing with macrophages with SMMC-7721 cells increased the oxaliplatin resistance by 0.4%, 2.0%, 4.6% and 9.0% respectively, compared with the control cells. Similarly, co-culturing macrophages with Huh-7 cells markedly increased the percentage of surviving cells by 4.7%, 5.4%, 10.2% and 13.4% respectively, when compared with control cells (Fig. 2b). Induction of apoptosis by oxaliplatin was further evaluated by annexin V staining. As presented in Fig. 2c, co-culturing with macrophages significantly decreased the proportion of annexin V positive SMMC-7721 (2.0% and 5.6%) and Huh-7 (5.3% and 12.8%) cells under oxaliplatin treatment.

### Co-culturing HCC cells with macrophages activate autophagy in HCC cells

**Fig. 3:**
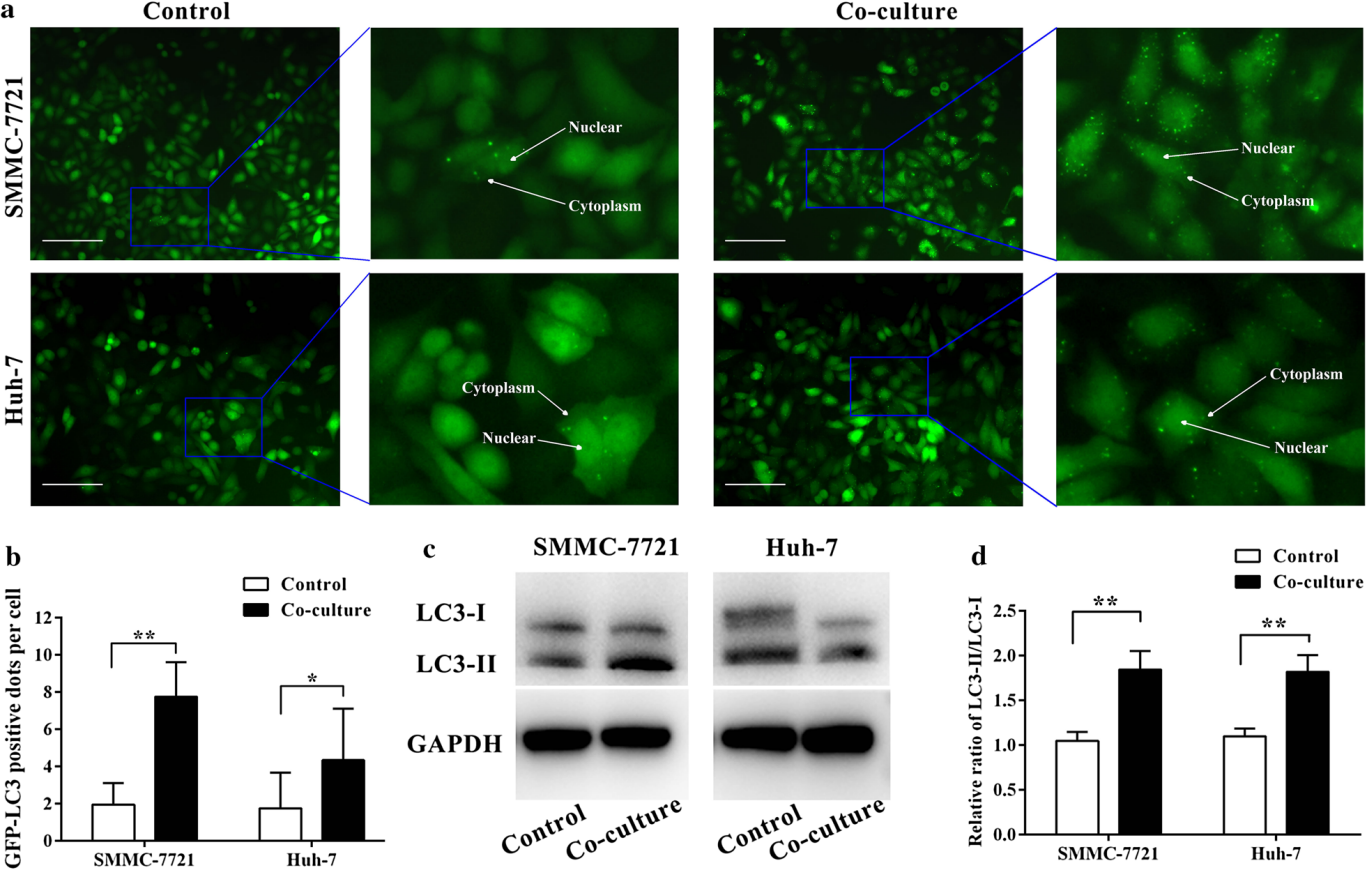
Co-culturing with macrophages activate autophagy in HCC cells. **a**, **b** SMMC-7721 and Huh-7 cells stably expressing the GFP-LC3 fusion protein were established and then respectively co-cultured with macrophages. GFP-LC3–positive dots per transfected cell were determined in 3 independent experiments. Eight random fields (×200) representing 200 cells were counted. Data shown are mean (SD) from at least 3 independent experiments. Means were compared between two groups using unpaired, two-tailed Student’s t-test. * P < 0.05, ** P < 0.01. **c**, **d** Western blot analysis of LC3-I and LC3-II in SMMC-7721 and Huh-7 cells after co-culturing with macrophages. Scale bars, 100 μm

We successfully established SMMC-7721 and Huh-7 cells which could stably expressing the GFP-LC3 fusion protein. Cells expressing GFP-LC3 showed that after 12 h of co-culture with macrophages, the GFP-LC3 signals shifted from a diffuse cytoplasmic pattern to a dot-like membrane pattern, indicating the formation of autophagic vacuoles (Fig. 3a). Morphometric analysis of the GFP fluorescence images showed that compared to control cells, there were obviously more GFP-LC3–positive dots per cell in SMMC-7721 and Huh-7 cells co-culturing with macrophages (Fig. 3b). Moreover, one vital characteristic of autophagy is autophagosome-associated form (LC3-II) which was the conversion of the soluble form of LC3 (LC3-I) to the lapidated. Consistently, western blotting data showed that co-culturing with macrophages increased the conversion of LC3-I into LC3-II in SMMC-7721 and Huh-7 cells (Fig. 3c, d).

### Autophagy inhibition partly abolish the oxaliplatin-resistance in HCC cells by co-culturing HCC cells with macrophages

**Fig. 4:**
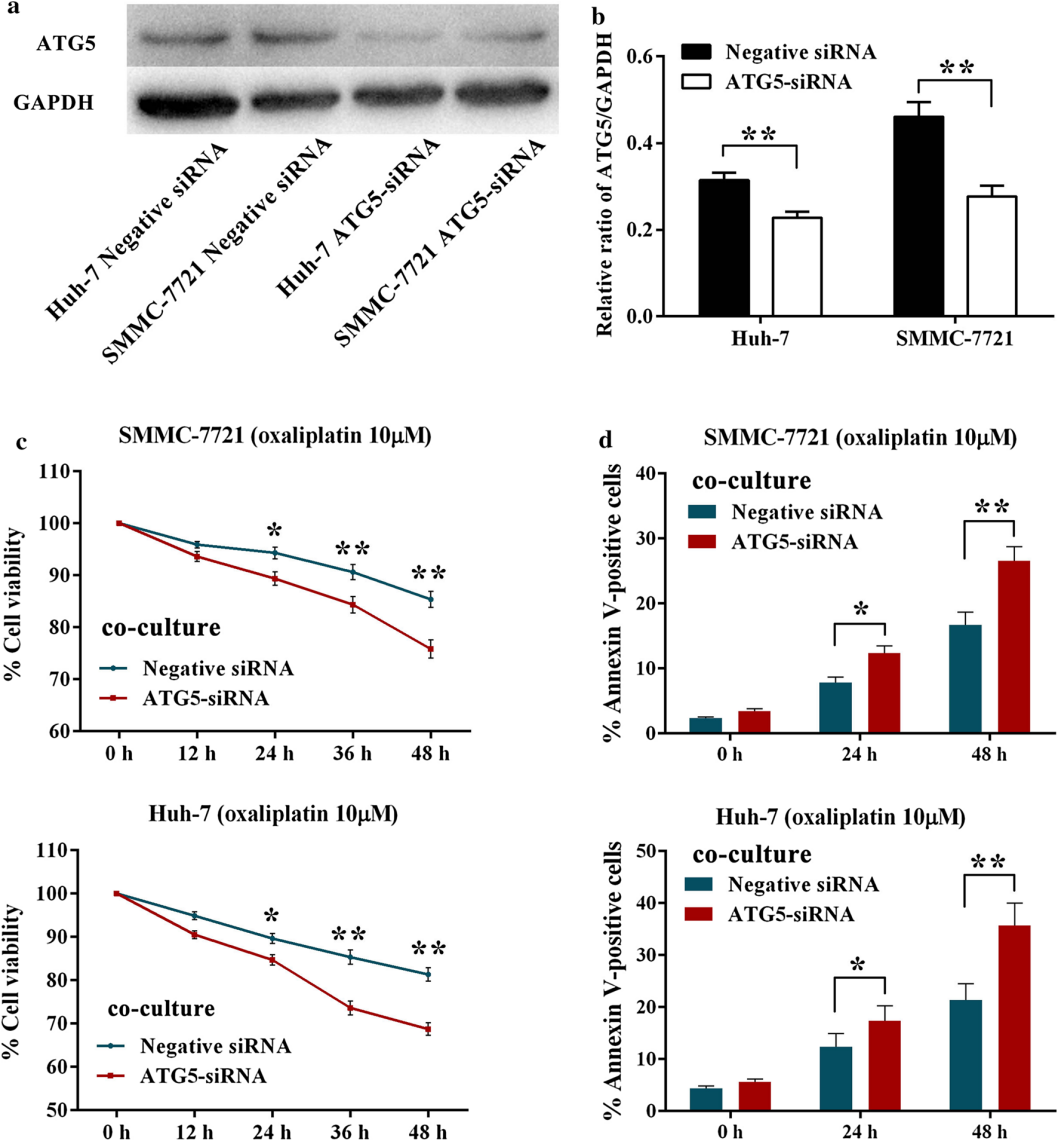
Autophagy inhibition partly abolish the oxaliplatin-resistance by co-culturing HCC cells with macrophages. **a**, **b** Western blot analysis of ATG5 conversion in SMMC-7721 and Huh-7 cells which transfected with Negative siRNA or ATG5-siRNA. **c**, **d** After transfected with Negative siRNA or ATG5-siRNA, the macrophages co-cultured SMMC-7721 and Huh-7 cells were exposed to 10 μM oxaliplatin for 12–48 h. The percentage of survived cells was determined with MTT assay and The percentage of apoptotic cells was determined with Annexin V staining assay. Data shown are mean (SD) from at least 3 independent experiments. Means were compared between two groups using unpaired, two-tailed Student’s t-test. * P < 0.05, ** P < 0.01

To examine whether the increased oxaliplatin-resistance of HCC cells was induced by autophagy after co-culturing with macrophages, ATG5 siRNA was adopted to inhibit autophagy. Western blotting analysis showed that siRNA successfully down-regulated the expression of ATG5 protein in HCC cells (Fig. 4a, b). After co-culturing with macrophages, the ATG5 siRNA-transfected SMMC-7721 and Huh-7 cells exhibited notable increased susceptibility to oxaliplatin compared with negative siRNA-transfected cells. As indicated in Fig. 4c, co-culturing with macrophages with ATG5 siRNA-transfected SMMC-7721 cells decreased the oxaliplatin resistance by 2.3%, 4.9%, 6.3% and 9.5% respectively, compared with the negative control cells. Similarly, co-culturing with macrophages with ATG5 siRNA-transfected Huh-7 cells markedly decreased the percentage of surviving cells by 4.4%, 5.0%, 11.7% and 12.6% respectively, when compared with control cells. Induction of apoptosis by oxaliplatin was further evaluated by annexin V staining. As presented in Fig. 4d, silencing of ATG5 significantly increased the proportion of annexin V positive SMMC-7721 (4.5% and 9.9%) and Huh-7 (5.0% and 14.3%) cells under oxaliplatin treatment.

### Co-implantation HCC cells with macrophages compromise the antitumor activity of oxaliplatin in HCC xenograft models

**Fig. 5:**
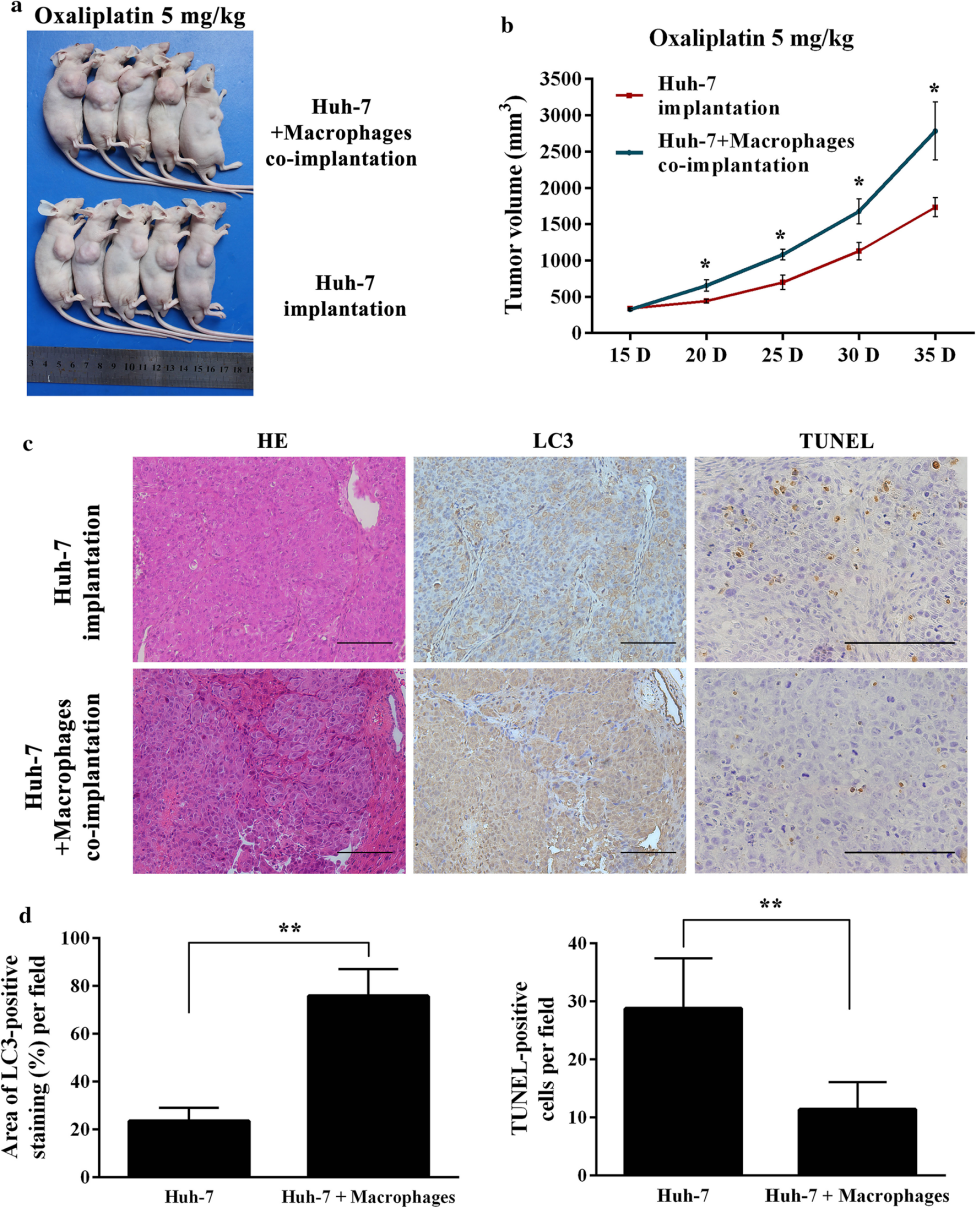
Co-implantation with macrophages compromise the antitumor activity of oxaliplatin in HCC xenograft models. **a** Tumor of mice from each group on 35 days after implantation are shown. **b** The volume of xenograft was calculated by a caliper every 5 days. Data shown are mean (SD). Means were compared between two groups using unpaired, two-tailed Student’s t-test. * P < 0.05. **c** The hematoxylin–eosin (×200), LC3 (×200) and TUNEL (×400) staining of xenografts tissue sections. **d** TUNEL-positive cells were counted manually in 8 randomly selected fields from each tumor sample. The area of the positive staining of LC3 in each photograph was measured by the Image-Pro Plus Software v6.2, and plotted as the percentage of photograph field area. Data shown are mean (SD) from at least 3 independent experiments. Means were compared between two groups using unpaired, two-tailed Student’s t-test. ** P < 0.01. Scale bars, 100 μm

In order to determine the in vivo relevance of our findings, we further investigated whether macrophages co-implantation can compromise the anticancer activity of oxaliplatin in Huh-7 derived tumor xenografts. Huh-7 or Huh-7 with THP-1 derived macrophages were respectively injected subcutaneously in nude mice, formed tumors in both groups, then received oxaliplatin treatment. At the 35th day, xenografts of Huh-7 with THP-1 derived macrophages were significantly larger (Fig. 5a), compared with xenografts of Huh-7 (2.784 ± 0.892 mm^3^ vs. 1.734 ± 0.892 mm^3^; P = 0.037). As shown in Fig. 5b, there were significant differences in tumor volume between two groups from the 20th day. Immunostaining of HCC cells in xenografts revealed higher expression of LC3 and a decreased of TUNEL-positive tumor cells in group of Huh-7 with THP-1 derived macrophages (TUNEL-positive cells per hpf: 28.8 ± 8.6 vs. 11.4 ± 4.7; P = 0.004), partially suggesting that macrophages co-implantation can induce autophagy in vivo to avoid apoptosis induced by oxaliplatin (Fig. 5c, d).

## Discussion

For unresectable HCC, TACE is a well recommended treatment, and it involves catheterization of the tumor supplyinig artery, followed by injection of embolization agents and chemotherapeutics through the catheter [5]. However, embolization is not always complete, and HCC is generally resistant to chemotherapy. Oxaliplatin is one of the most widely used chemotherapeutic drugs in liver cancer interventional therapy, and oxaliplatin-based chemotherapy is now the most commonly used chemotherapeutic criterion in HCC [26]. Unfortunately, inoperable HCC patients who develop resistance to oxaliplatin have limited optimal therapeutic strategy [7]. Our results showed that the density of TAMs in HCC patients with tumor shrinkage after TACE was greater than that in patients without tumor shrinkage. This phenomenon suggested that the macrophages infiltrated in tumor may be associated with drug resistance.

In addition to genetic changes of HCC cells themselves, tumor microenvironment has an important role in chemo-resistance [27, 28]. In the context of chemotherapy treatment, TAMs have become effective regulators of therapeutic response [10, 11]. These effector cells can regulate tumor cell survival pathways by providing of cytokines and pro-tumorigenic proteases [17, 29]. Additionally, TAMs can suppress immune-based mechanisms of cytotoxic chemotherapy [30, 31]. In the present study, we showed that the HCC cells exhibited enhanced resistance to oxaliplatin after co-culturing with macrophages in vitro and vivo, indicated that TAMs can induced chemo-resistance in HCC.

Autophagy serves as a dynamic recycling system that produces new building blocks and energy for cellular homeostasis and renovation [32]. It has been demonstrated that autophagy can protect cancer cells against hypoxia, metabolic stress, detachment-induced anoikis and diverse cellular damages, as well as apoptosis or necrosis induced by anti-tumor therapy or other cell death stimuli [33–37]. To directly determine the levels of autophagy occurred in SMMC-7721 and Huh-7 after co-culturing with macrophages, the GFP-LC3 redistribution was observed. The elevated number of GFP-LC3–positive dots per cell in oxaliplatin-treated cells indicated that co-culturing with macrophages could activate autophagy in HCC cells. Similarly, western blotting analysis of LC3 conversion in HCC cells also proved this conclusion. To directly determine the role of autophagy induced by macrophages, RNA interference of the essential autophagy gene ATG5 was used to inhibit autophagy in HCC cell. After co-culturing these HCC cells with macrophages, increased oxaliplatin cytotoxicity was observed in ATG5 siRNA-transfected HCC cells. Moreover, the decreased number of TUNEL-positive HCC cells in THP-1 derived macrophages and HCC cells blended xenografts with simultaneously high level of LC3 expression suggests that macrophages may decrease the anticancer effect of oxaliplatin in vivo by activation of autophagy. These findings indicated that autophagy induced by macrophages in HCC cells may play a vital role in resistance to chemotherapy agents.

There are some limitations to our study. First, TACE is a form of intra-arterial catheter-based chemotherapy that selectively delivers high doses of cytotoxic drug to the tumor bed, combined with the effects of ischemic necrosis induced by arterial embolism. Therefore, we cannot ignore the effect of hypoxia induced by TACE on chemo-resistance. Second, because of the non-contact co-culture system in our study, macrophages may induce autophagy in HCC cells by secretion of certain cytokines. But in vivo, macrophages and HCC cells are in direct contact. Our cell co-culture model cannot perfectly simulate the heterologous and tumor cells in HCC, and more effort should be made to clarify how the macrophages induce autophagy in HCC cells. Third, the detection of cell apoptosis by Annexin V test at the time of no intervention of oxaliplatin involves a problem of spontaneous apoptosis of the cells. Actually, we indeed found that the apoptosis rate of HCC cells at time 0 h oxaliplatin treatment was slightly reduced in co-culture group during the research. The reason we believe in may be associated with the enhanced autophagy or secreted anti-apoptotic factors by co-culturing with macrophages, leading to a decrease in the rate of spontaneous apoptosis. Moreover, the difference in apoptotic rates become more significantly after oxaliplatin treatment. Therefore, in this study, we focused on the reactivity of HCC cells to oxaliplatin before and after co-culture with macrophages. Last, our results only revealed that macrophages could enhance chemo-resistance of HCC cells by activating autophagy in HCC cells. However, TME includes surrounding blood vessels, immune cells, fibroblasts, signaling molecules and the extracellular matrix. Recent studies have shown that the stromal cells in HCC can regulate the response of cancer cells to chemotherapy [38–40]. We hypothesized that macrophages in TME may be affected by other heterogeneous cells in vivo, so further studies to reveal the mechanism will be necessary conducted.

## Conclusions

In summary, macrophages may induce autophagy in HCC cells which contributes to drug resistance and inhibition of autophagy potentiates oxaliplatin cytotoxicity. Targeting TAMs will be a promising therapeutic strategy to enhance the effects of chemotherapy and improve clinical outcomes in HCC patients.

## Abbreviations

HCC: hepatocellular carcinoma
TME: tumor microenvironment
TAMs: tumor-associated macrophages
TACE: transarterial chemoembolization
ROS: reactive oxygen species
FBS: fetal bovine serum
PMA: phorbol myristyl acetate
PI: propidium iodide
PBS: phosphate-buffered saline
H&E: hematoxylin and eosin
TUNEL: terminal deoxynucleotidyl transferase-mediated deoxyuridine triphosphate nick-end labeling
TdT: terminal deoxynucleotide transferase
